# Hepatitis outbreak in children: Medico-legal implications and liability protection. Have we learned anything from the COVID-19 pandemic?

**DOI:** 10.3389/fpubh.2022.989433

**Published:** 2022-08-11

**Authors:** Andrea Cioffi, Camilla Cecannecchia

**Affiliations:** Department of Anatomical, Histological, Forensic and Orthopaedic Sciences, Sapienza University of Rome, Rome, Italy

**Keywords:** infectious disease, professional liability, legal claims, causation, public health

## Introduction

Based on data provided by the World Health Organization (WHO), since 5 April 2022, 650 cases of severe hepatitis of unknown origin in children have been reported in America and Europe (data of 26 May 2022). The cases concern children aged between 1 month and 16 years. Thirty-eight children (about 6%) needed a liver transplant; in addition, at least nine children died. The pathology is manifested in most cases with nonspecific gastrointestinal symptoms (abdominal pain, nausea, vomiting, diarrhea) followed by jaundice and a significant increase in liver enzymes (aspartate transaminase (AST) or alanine aminotransferase (ALT) greater the 500 IU/L). The etiopathogenesis of this form of acute hepatitis is still unknown: in all known cases the presence of the most common hepatotropic viruses (Hepatitis Viruses A, B, C, D and E) has been ruled out. Adenovirus, more frequently F type 41, was found in 74 cases. Of the cases tested for SARS-CoV-2, 20 were positive and 19 showed SARS-CoV-2 and Adenovirus co-infection (1). To date, Adenovirus infection is the most likely cause. Nevertheless, it is specified that Adenovirus infection - more frequently responsible for gastroenteritis, cystitis and conjunctivitis (2) - would not explain the prognostic severity of this new form of acute hepatitis in healthy children. In addition, Adenovirus type F41 is generally not responsible for acute hepatitis in non-immunodepressed children (3, 4). Finally, it is not yet clear whether SARS-CoV-2 may have a causal or concausal role in the etiopathogenesis of pathology. It is not even definitive the hypothesis of an infectious pathology; in fact, the cause of this hepatitis could also be non-infectious. What is certain is that today this form of hepatitis is manifested in children of all ages, with non-specific symptoms and signs and is associated with a prognosis varying from complete healing to death.

Several countries have issued recommendations to diagnose new cases of fulminant hepatitis of unknown origin in children and to properly track and report them to health authorities. This precautionary measure is essential to isolate the infectious disease as much as possible and reduce the risk of spread. Therefore, in the presence of a young patient with gastrointestinal symptoms—which are non-specific and very frequent in children—health care professionals are required to exclude the presence of this new form of hepatitis through further tests (such as transaminase levels and molecular research of adenoviruses). However, even in the case of diagnostic suspicion, there are no useful indications for the management and treatment of affected patients. Therefore, as in all diseases of unknown origin and new onset, health personnel are forced to resort to empirical therapy, i.e., to start treatment before the confirmation of the disease and in the absence of complete information on its etiology. Therefore—in the absence of guidelines and/or operational indications—healthcare professionals, in managing such patients by attempting the best possible therapeutic approach, will be able to rely exclusively on their professional experience and the specific clinical evolution of the individual patient. In fact, treatment, to date, is supportive and based on patient hydration and body temperature control (3–5). However, as already stated, this therapeutic strategy is not standardized and is associated with extremely variable clinical responses for reasons still unknown.

## Medico-legal implications

This situation, in the terminal phase of the health emergency related to the COVID-19 pandemic, once again exposes both the individual health worker and the health facility to the risk of medical-legal litigation in both civil and criminal contexts (6, 7). In fact, the difficulty in diagnosing this hepatic infectious disease—in addition to causing a therapeutic delay—could delay the isolation of the patient, and thus, expose the population to the risk of contagion and consequent health risks. In contrast, the treatment—since it is not based on guidelines and/or recommendations—is entrusted to the direct experience of the single doctor and is, therefore, by definition, susceptible to unpredictable outcomes. This unpredictability is characteristic of all newly-emerging infectious diseases—especially if diffusive—for which there is no scientific evidence to support the work of healthcare professionals. The risk of medical-legal disputes in such types of pathologies is particularly high, as demonstrated by the experience of the COVID-19 pandemic (6–9). Indeed, during the COVID-19 pandemic, questions arose about legal liability risks for physicians and other health care providers thrown into this uncertain clinical environment. So, from the medico-legal point of view, the main question was: “How could they be shielded from liability while preserving patients' rights to sue for damages?” (10). In addition, the need for legal shield has proved necessary both for physicians treating COVID-19 patients in a volunteer capacity and for non-volunteer physicians (11).

Therefore, the end of the “health pandemic” by COVID-19 risks being succeeded by a real “judicial pandemic,” In fact, based on our experience, in Italy, there is a progressive and worrying increase in the number of claims for damages related to SARS-CoV-2 infection due to alleged civil liability of healthcare personnel and/or healthcare facilities. On the contrary, the same increase is not found in the criminal sphere; this is due to the Italian law 76/2021 (12) that, from June 1, 2021, guaranteed a criminal legal protection in Italy to healthcare professionals who faced cases of COVID-19. This measure introduced the so-called “criminal shield” that excludes the punishability of healthcare professionals in case of culpable homicide and culpable personal injuries resulting from the exercise of their profession in the healthcare emergency phase.

Also, regarding tort law, may be necessary - for this type of emergency situations - a “no fault system.” In this way we could set a standard compensation with a maximum economic amount for the damage suffered in case of pathologies of unknown and/or new origin. This could avoid undermining the economic stability of National Health Systems (13).

The current amount of medical-legal litigation has as its object the presumed incorrect diagnostic-therapeutic management by healthcare personnel of a diffusive infectious disease that, especially in the first phase of the pandemic, was completely unknown. Like the new pediatric hepatitis, the SARS-CoV-2 infection also required the isolation of the affected patient and an empirical diagnostic-therapeutic procedure not based on known scientific evidence.

## Discussion

Medical-legal disputes are particularly fertile ground in the case of diffuse infectious diseases such as COVID-19 and acute hepatitis of unknown origin in children.

It is important to note that during the early stages of the COVID-19 pandemic front-line doctors faced complex and new clinical challenges in the absence of guidelines, scientific evidence, and expertise in this specific field (13). In addition, during the first stages of the health emergency, national and international recommendations were issued without adequate rigor and high specificity since, obviously, were the result of research still in the embryonic stage since the pathology studied was totally new (13). In addition to the scarcity of reliable scientific evidence, another problem - with potential civil and criminal medico-legal implications - was related to the fact that non-specialist doctors and resident doctors were also recruited to cope with the health emergency (13, 14).

Therefore, especially in the light of the lessons learned from the pandemic experience and the increasing prevalence of infectious diseases, it would be advisable to first formulate an agreed definition of “unknown infectious disease,” This definition should be based on the standard criteria for defining a scientifically unknown disease, such as a disease not known from international literature, an unknown spreading disease with a high risk of transmission, a disease with an undefined etiology, a disease that can only be diagnosed based on exclusion criteria, a disease for which there is no specific treatment, a disease associated with unpredictable outcomes and a variable prognosis.

A specific standardized protocol should be combined with an *ad hoc* measure to protect healthcare workers. In fact, legal bodies should guarantee a civil and criminal shield for healthcare personnel and facilities directly involved in the management of the “unknown infectious disease.” A legal shield would be justified by the total unpredictability of potential outcomes on individual and public health. It should be imperative to automatically protect healthcare personnel from the earliest stages of the spread of the disease. In fact, precisely in the initial stages of a health emergency—characterized by operational opinability—health personnel are significantly exposed to the risk of medical-legal disputes.

In the medico-legal and juridical field, a standardized definition of “unknown infectious disease” would be appropriate; consequently, in the event of the occurrence of an unknown pathology, it will be crucial to automatically establish a criminal and civil shield that protects the front-line healthcare personnel. We believe that this medical-legal protocol could be a useful tool not only to reduce medical-legal disputes (associated with huge economic losses for the health system) but also to prevent the spread of “defensive medicine” (15). In fact, in managing (without appropriate protection) pathologies that are new in the scientific panorama, healthcare professionals could be inclined to make professional decisions based on the possible judicial consequences, rather than on the actual health advantage of the individual patient. Therefore, the use of a protocol similar to the one we propose would also be a valid tool to maintain high-quality standards in the management of the patient affected by an unknown infectious disease. The legal shield in the case of “unknown infectious disease” should be applicable to all medical specialties (not only for doctors specializing in infectious diseases); in fact, especially in cases of pandemic evolution, doctors with various expertise and specializations could be recruited. Therefore, a homogenization of the applicability of the legal shield would be necessary in order to protect all the physicians in front line in the contrast of an infectious pathology of unknown origin. In parallel with a specific legal shield for the “unknown infectious diseases,” Schaffer et al. (16) have proposed a useful decision-making framework, usable by individual physicians, where they consider their personal and professional obligations in regard to resource stewardship, innovation in practice, patient-specific contexts, and patient advocacy while acting outside of their speciality.

To identify the situations in which it would be appropriate to apply the legal shield and to better organize the management of infectious diseases of unknown origin (both within health facilities and on the territory), it might be useful to use a specific technical support framework (STSF), already used in some countries. Specific protocols and recommendations that can be used in healthcare could be implemented to optimize care. A doctor who will follow the specific recommendations will be able to take advantage of the legal shield; on the contrary, it will be appropriate to compensate any damage resulting from errors (17).

In addition, it will be important, worldwide, to implement risk management specific programs to better manage the possible medico-legal consequences related to these emergency situations. A system that can be used as a model may be that of the U.S.A., in which the patient safety and quality of care are focal (18).

Finally, in the case of hepatitis of unknown origin in children, it should be pointed out that not all cases are of unknown origin. In fact, it would be incorrect to identify a certain causal correlation for all new cases of hepatitis in children. From a medico-legal perspective, it is not correct to attribute all cases of hepatitis in children to this new unknown form. In fact, it will be essential to respect medico-legal criteria that foresee the exclusion of all other possible causes before being able to state that hepatitis in a child is indeed “of unknown origin.” Not to incur in this error is fundamental to manage adequately, from the clinical point of view, all cases; above all, it is crucial to activate an adequate national and international health surveillance with a possible suitable application of the criminal and civil shield.

The absence of a legal shield could also worsen the quality of care; although, to date, there is insufficient evidence that liability protection could affect medical performance, it is likely that the increase in stress - induced by the fear of doctors being prosecuted for mistakes made during their activity - worsens the quality of medical care (19). In the case of pathologies of unknown origin, the limitation induced by defensive medicine could discourage some doctors in participating, even resorting to diagnostic and treatment attempts (indispensable in facing pathologies of unknown origin) the timely definition of specific treatments. Moreover, the absence of a legal shield could lead to a dangerous passivity in the activities of front-line physicians, resulting in an increased risk of bad outcomes in patient management.

In conclusion, the strengths of applying a legal shield in case of 'unknown infectious disease', are the protection of doctors at the forefront in the battle against new and unknown diseases resulting in greater serenity of the individual doctor in his professional activity and likely improvement of the quality of care (also according to the principle of reciprocity). In addition, lawsuits for damages brought without scientific and medical-legal basis could be reduced.

The weakness of a possible application of a legal shield is the risk that patients who should actually be compensated for damages resulting from gross medical errors will not be protected. In addition, it should be avoided that healthcare facilities take advantage of the legal shield to cover structural deficiencies. The possible solutions to these two problems are, respectively, the correct formulation and application of protocols created *ad hoc* by the STSF, and the creation of a legal shield directed only to individual doctors and not to health facilities.

## Author contributions

AC conceived the idea, revised, and edited the manuscript. CC drafted the manuscript. Both authors read and approved the final version of the article.

## Conflict of interest

The authors declare that the research was conducted in the absence of any commercial or financial relationships that could be construed as a potential conflict of interest.

## Publisher's note

All claims expressed in this article are solely those of the authors and do not necessarily represent those of their affiliated organizations, or those of the publisher, the editors and the reviewers. Any product that may be evaluated in this article, or claim that may be made by its manufacturer, is not guaranteed or endorsed by the publisher.
